# Development and validation of the body image scale for youth (BISY)

**DOI:** 10.1186/s40337-022-00657-z

**Published:** 2022-09-06

**Authors:** Sara Jalali-Farahani, Parisa Amiri, Fariba Zarani, Farid Zayeri, Fereidoun Azizi

**Affiliations:** 1 Research Center for Social Determinants of Health, Research Institute for Endocrine Sciences, Shahid Beheshti University of Medical Sciences, Tehran, Islamic Republic of Iran; 2 Department of Clinical and Health Psychology, Faculty of Psychology and Educational Sciences, Shahid Beheshti University, Tehran, Islamic Republic of Iran; 3 Department of Biostatistics, Proteomics Research Center, Faculty of Allied Medical Sciences, Shahid Beheshti University of Medical Sciences, Tehran, Islamic Republic of Iran; 4 Endocrine Research Center, Research Institute for Endocrine Sciences, Shahid Beheshti University of Medical Sciences, P.O.Box: 19395-4763, Tehran, Islamic Republic of Iran

**Keywords:** Body image, Adolescents, Validity, Reliability, Body weight status

## Abstract

**Background:**

Body dissatisfaction has been known as an important public health concern that can influence the physical and psycho-social health of adolescents. Hence, health professionals need a valid and reliable tool to assess this problem and its related factors in youth. This study aims to develop and assess the psychometric properties of an indigenous scale for the evaluation of body image and to investigate its association with body weight status among Iranian adolescents.

**Methods:**

This study was conducted on 857 adolescents who completed the body image scale for Youth (BISY) and self-reported their body weight and height. Face, content, and construct validity methods were used to assess the validity of the BISY. Exploratory factor analysis (EFA) was used to assess construct validity. Moreover, the internal consistency of the scale was assessed by calculating Cronbach’s alpha coefficient (α), and test–retest reliability was examined using the intra-class correlation coefficient (ICC). To compare the BISY scores across sex and body weight status groups, the Independent samples t-test and the analysis of variance (ANOVA) or Welch tests were used, respectively.

**Results:**

Mean age and body mass index (BMI) of participants were 16.5 ± 1.1 years and 22.4 ± 4.5 kg/m^2^, respectively. The EFA showed a 10-factor construct which explained 57% of the variance. The Cronbach’s alpha coefficient for overall items of the scale was 0.896 and ranged from 0.42 to 0.92 for subscales. The test–retest reliability result was acceptable for the BISY (ICC = 0.805). The ICC values ranged from 0.753 to 0.990 for BISY subscales. The BISY total score was significantly higher in girls compared to boys (34.8 ± 12.7 vs. 31.3 ± 10.2; *p* < 0.001). In girls, the BISY total score was significantly lower in normal-weight girls compared to their overweight (*p* = 0.009), and obese (*p* = 0.012) counterparts. This difference was not observed in boys.

**Conclusion:**

These findings support the reliability and validity of the BISY for the assessment of body image in Iranian adolescents; this scale can be applied as an appropriate tool for the assessment of body image in adolescents in related future studies. Current findings highlight considering body image as an important aspect of intervention programs targeting overweight and obesity in adolescents, specifically girls.

**Plain English summary:**

Negative body image has been known as an important public health concern that can adversely influence different aspects of adolescents’ health. Hence, a valid and reliable tool is necessary to identify adolescents at risk of developing negative body image and its related disorders. There is a lack of an indigenous scale that can comprehensively assess body image and its related factors in Iranian adolescents; hence, the current study aimed to develop and assess the psychometric properties of an indigenous scale for evaluation of body image and further investigate the association between body image and body weight status among Iranian adolescents. The current findings support the reliability and validity of the BISY. In addition, more negative body images were observed in girls compared to boys and in overweight/obese girls compared to their normal-weight counterparts. Therefore, promoting a healthy body image should be incorporated as an important component of future health promotion programs to address obesity, eating disorders, and other health-related concerns among adolescents, specifically girls.

**Supplementary Information:**

The online version contains supplementary material available at 10.1186/s40337-022-00657-z.

## Introduction

Body image is an important issue during adolescence; pubertal and identity development during this stage make it a critical period for the formation of negative body image [1]. A negative body image may adversely influence physical, psychological, and social aspects of adolescents’ health; as findings of previous studies have frequently demonstrated its association with the risk of obesity, eating disorders, suicide ideation, low self-esteem, and depressed mood in youth [2–6]. Moreover, negative body image found to adversely influence adolescents’ social interactions with their parents and peers [7–9]. Therefore, it is important to identify those adolescents who are at risk of developing body image disturbances.

To assess different aspects of body image, various scales including the Body Shape Questionnaire (BSQ), the Drive for The Muscularity Scale (DMS), the Adolescent Body Image Satisfaction Scale (ABISS), the Body Image Coping Strategies Inventory (BICSI), the Body image scale (BIS), the Multidimensional Body-Self Relations Questionnaire-Appearance Scales (MBSRQ-AS), and the Eating Attitudes Test (EAT-26) have been developed [10–16]. Although several tools exist for the evaluation of body image; most of them were designed to assess the certain dimension of body image. For example, the Body Shape Questionnaire (BSQ) is a 34-item scale that measures body shape concerns that can associate with the development, maintenance, and treatment of eating disorders [10]. The Drive for The Muscularity Scale (DMS) is another scale that focused on attitudes and behaviors related to muscularity. This scale expresses the extent an individual is preoccupied with increasing muscularity and a higher score show more drive for muscularity [11]. The 16-item Adolescent Body Image Satisfaction Scale (ABISS) has been developed to assess body image satisfaction in male adolescents and encompasses three subscales of body competence, body inadequacy, and internal conflict [12]. The Body Image Coping Strategies Inventory (BICSI) is a 29-item instrument that was developed for the assessment of three main coping strategies including avoidance, appearance fixing, and positive rational acceptance which are used for the management of threats or challenges related to an individual’s body image experiences [13]. The Multidimensional Body-Self Relations Questionnaire-Appearance Scales (MBSRQ-AS) is a 34-item instrument that covers several dimensions of body image and includes five subscales: appearance evaluation, appearance orientation, overweight preoccupation, self-classified weight, and body areas satisfaction scale. It is one of the most comprehensive instruments that is widely used across different countries [16]. The Body image scale (BIS) is a 35-item instrument that has been developed for assessment of body dissatisfaction in young adult female in Pakistan [14]. This scale encompasses three main subscales including physical component, psychological component, and strategies used to maintain one’s body image [14]. Most of these instruments have emphasized on evaluation of a unique dimension of this concept including concerns, attitudes and behaviors, satisfaction, or coping strategies related to an individual’s body image. Past studies conducted on Iranian adolescents with a focus on body image topic, have also been confined to measurement of certain components of body image such as body dissatisfaction, body image coping strategies, and eating disorders [17–19]. Since, body image is a multi-dimensional constructs and there is consensus about the multi-dimensional properties of this concept by body image scholars [20–22]; developing a comprehensive tool covering various dimensions of body image can help experts to better understand and assess this concept in related research.

Previous studies endorsed a significant association between individuals’ perceptions regarding their bodies and the cultural context of society [23–25]. There are significant differences in cultural values of Asian and Western countries as well as east and west Asian cultures. For instance, in Asian countries, obesity was considered a sign of health and wealth, and thinness was considered to indicate weakness and illness [26]. Moreover, the Islamic law regarding hijab in west Asian countries like Iran, such as requiring the covering of the body, especially in females, may impact body parts that occupy the females' minds and consequently have an influence on the preoccupation with the body shape and size [27]. These differences, limit the appropriate use of existing tools for the assessment of body image in a west Asian country like Iran. Since, most of the existing scales are developed in other countries, mainly Western ones [10–13, 15, 16], they cannot be favorably applied to our indigenous population. Therefore, considering the lack of a comprehensive and indigenous tool for the assessment of body image in Iranian adolescents; developing such a scale seems essential. Hence, the current study aimed to first, develop and assess psychometric properties of an indigenous scale for evaluation of body image in adolescents. Then, its second aim was to investigate the association between body image and body weight status among a sample of Iranian adolescents.

## Methods

### Participants

A total of 903 adolescents (aged 15–18 years) residing in Tehran participated in the current study. According to the previous studies, some physical illnesses that have visible impacts on the body and those that negatively influence body functions can adversely affect body evaluation and body dissatisfaction [28]. Furthermore, some mental disorders are associated with weight gain due to lifestyle changes, and the side effects of medications used to treat some severe mental health problems, all of which can affect people's perception of their body image [29, 30]. Therefore, in the current study, a total of 46 adolescents were excluded from the analysis due to having chronic mental or physical diseases (such as depression, social anxiety, anxiety and stress, convulsion, cancer, diabetes, heart diseases, kidney diseases, and thyroid disorders); hence, data of 857 adolescents were analyzed.

Before data collection, ethical approval was obtained from the Ethics Committee of the Research Institute for Endocrine Sciences (RIES) affiliated with Shahid Beheshti University of Medical Sciences, Tehran, Iran. Furthermore, the approvals were obtained from the Ministry of Education in Tehran and selected high schools. All participants provided written informed consent.

### Measurements

Participants were asked to answer a set of questions regarding socio-demographic variables such as their age, biological sex, and history of diseases as well as their parent's marital status, level of education, and job status and self-report their weight, and height. Body mass index (BMI) was calculated as weight (kg) divided by the square of height (m^2^). The BMI-for-age national percentiles were used to determine the body weight status of adolescents. Underweight, normal weight, overweight, and obesity were defined as BMI values of < 5th percentile, ≥ 5th to < 85th percentile, ≥ 85th percentile to < 95th percentile, and ≥ 95th percentile, respectively [31]. In addition, participants were also asked to fill out the Eating Attitude Test-26 (EAT-26), and body image scale for Youth (BISY). The EAT-26 questionnaire is a 26-item scale encompassing three subscales including (1) dieting, (2) bulimia and food preoccupation, and (3) oral control. A higher score of EAT-26 indicates a higher risk for the development of eating disorders [15]. Furthermore, participants were asked to self-assess three aspects of their physical bodies’ features. For this purpose, participants rated their physical appearance, physical ability, and physical health from 0 to 20.

### Development and scoring of the BISY

The initial item pool consisted of 95 items that were developed by the authors based on the findings of a qualitative study and a review of existing literature on body image topic. Ten themes were identified using the findings of this qualitative study including (1) personal characteristics and strategies, (2) priority of health and spirituality, (3) appearance importance in the future, (4) appearance importance in social interactions, (5) social models, (6) perceived cultural values, (7) perceived social support, (8) empowerment, (9) body evaluation, (10) emotions and behaviors. The part of these findings with a focus on the psychological aspects has been published earlier [32]. For all subscales except for “emotions and behaviors”, a five-point Likert scale from one to five was used for scoring answers for each item, where the choice of an answer for “completely agree” was given a five-point and “completely disagree” was given one point for items belonging to “social models”, “appearance importance in social interactions”, and “perceived cultural values” subscales. The remaining items belonging to other subscales are scored reversely. For the “emotions and behaviors” subscale, the choices ranged from “always” to “never” with values of five and one for always and never, respectively. Then for better interpretation, the 1–5 point scale items are transformed to 0–100 as follows: 1 = 0, 2 = 25, 3 = 50, 4 = 75, and 5 = 100. To calculate subscale and total scores of the BISY, the mean is computed as the sum of the items over the number of items. Hence, the scores ranged from 0 to 100. A higher total score indicates a more negative body image.

### Validity assessment

The validity of the scale was assessed using face, content, and construct validity methods as described below.

1. Face validity

Face validity was assessed using qualitative and quantitative methods. In the qualitative method, a total of ten adolescents were asked to assess difficulty, relevancy, and ambiguity on the preliminary scale. In the quantitative face validity method, the same participants were asked to rate each item on the scale by the 5-point Likert scale from completely important (score 5) to not at all important (score 1). Then, the item impact score was calculated using the below formula:

$${\text{Item impact score}} = {\text{frequency }}\left( \% \right) \times {\text{importance}}$$

Frequency (%) indicates the number of participants who gave the item a score of 4 or 5. Items with an impact score of more than 1.5 were considered appropriate and maintained for the next stage.

(2) Content validity

Content validity of the scale was assessed by a panel of experts in different disciplines including health education, community nutrition, sociology, psychology, and medical sciences. For qualitative content analysis, experts were asked to comment on the style, wording, and scoring of the items. In addition, for quantitative content analysis, content validity ratio (CVR) and content validity index (CVI) were calculated. To calculate CVR, firstly, each item was scored using a 3-point Likert scale (essential, useful but not essential, not essential) by each expert. Then, using the below formula the CVR was calculated:

$$CVR=\frac{\mathrm{ne}-\mathrm{N}/2}{\mathrm{N}/2}$$

In this formula, N is the total number of expert panels and ne is the number of individuals who considered the relevant item “essential”. Using Lawshe’s table, the CVR higher than 0.62 for 10 individuals (based on the number of experts in the current study) indicates the necessity of the item [33].

For calculating CVI, experts were asked to comment independently on the degree of the relevance, clarity, and simplicity of each item using a 4-point Likert scale (“not at all” to “completely”). Then, using the below formula CVI was calculated.

$$CVI=\frac{{\text{Number of raters chose point}}3{\text{ and }}4 }{{\text{total number of raters}}}$$

Scale’s content validity index (S-CVI) was calculated by taking the sum of all item CVIs divided by the total number of items.

(3) Construct validity

Construct validity of the scale was evaluated using exploratory factor analysis (EFA). For this purpose, the Principal component analysis with Varimax rotation was used. The Kaiser–Meyer–Olkin (KMO) and Bartlett Sphericity Test were used to show the sampling adequacy. The KMO value of ≥ 0.8 and the *p*-value for the Bartlett Sphericity test of < 0.05 indicate the sampling adequacy for EFA [34]. Scree plot, eigenvalues greater than 1, and the number of factors that explain > 50% of variance were used to predict the number of factors retained. In this analysis, items with factor loadings over cutoff values of 0.4 were considered important and remained in the model.

### Reliability assessment

The reliability of the scale was assessed using Cronbach's alpha coefficient (α) and intra-class correlation coefficients (ICCs).

1. Internal consistency

The Cronbach's alpha coefficient (α) was used to assess the internal consistency of the scale. The Cronbach's alpha coefficients were calculated for total and subscale scores of the BISY and α values greater than 0.7 were considered acceptable [35].

(2) Test–retest reliability

Test–retest reliability was determined using the intra-class correlation coefficients (ICCs). For this purpose, the scales were completed by 30 adolescents within a 10–14 days interval and intra-class correlation coefficients of total and subscale scores of the two tests were calculated, and values greater than 0.5 were considered acceptable [36].

### Data analysis

To analyze data, the SPSS software (version 21.0) was used. Descriptive statistics including mean ± sd for continuous variables and frequency (%) for categorical variables were reported. To investigate any significant differences in the distributions of categorical variables (adolescents’ body weight status and parent's marital status, levels of education, and working status) in sex groups, the Chi-square test was used. To compare total and subscale scores of the BISY between boys and girls, the Independent samples t-test was used. In addition, the analysis of variance (ANOVA) or Welch tests was applied to compare total and subscale scores of the BISY among body weight status groups. For the post hoc test, the LSD or Dunnett T3 tests were used. In addition, to determine the correlation between continuous variables including body image total score and EAT-26 scores as well as body image total score and body self-assessed features including physical appearance, ability, and health scores, Pearson correlation coefficients were reported. In all statistical analyses, *p*-values < 0.05 were considered significant.

## Results

### Descriptive statistics

The mean age and BMI of participants were 16.5 ± 1.1 years and 22.4 ± 4.5 kg/m^2^, respectively. Descriptive statistics for sociodemographic variables and the body weight status of study participants are presented in Table 1. There were no significant differences between boys and girls in terms of parental marital status, level of education, and working status. About half of both mothers and fathers had academic degrees. Most mothers were housewives and about one-third of fathers were employee and about half of them were self-employed. In terms of distribution of body weight status, there was a significant difference between boys and girls.

**Table 1:** Descriptive statistics of adolescents by sex groups

	Boys (n = 422)	Girls (n = 435)	*p* value*
*Marital status of parents*			
Married	403(95.5)**	406(93.3)	0.257
Divorced/widowed	19(4.5)	29(6.7)	
*Maternal level of education*			
Primary	59(14.0)	73(16.8)	0.267
Secondary	168(39.8)	152(34.9)	
Higher	195(46.2)	210(48.3)	
*Maternal working status*			
Housewife	314(74.4)	333(76.6)	0.466
Employed/student	108(25.6)	102(23.4)	
*Paternal level of education*			
Primary	78(18.5)	77(17.7)	0.771
Secondary	139(32.9)	136(31.3)	
Higher	205(48.6)	222(51.0)	
*Paternal working status*			
Employee/laborer	145(34.3)	164(37.7)	0.467
Self-employed	236(55.9)	225(51.7)	
Unemployed/retired	41(9.7)	46(10.2)	
*Adolescents’ body weight status*			
Underweight	11(2.6)	28(6.4)	< 0.001
Normal-weight	246(58.3)	308(70.8)	
Overweight	79(18.7)	63(14.5)	
Obese	86(20.4)	36(8.3)	

*P values were derived using the Chi-square test

**Data is shown as frequency (percentage)

### Validity and reliability assessments

In the quantitative face validity assessment, the importance of each item was assessed and items with an impact score of < 1.5 were eliminated. In this stage, one item was eliminated. In qualitative content validity assessment, a total of 29 items were deleted due to having overlaps with other items or assessing lots of unnecessary details. Another five items were deleted because they did not obtain acceptable CVI and CVR levels in the quantitative assessment of the content analysis.

Then, a total of 60 items entered the construct validity assessment. In EFA, another 8 items were removed due to having low values of communality or factor loading or both. For 52 items (Additional file 1: Table S1) that remained in EFA, the KMO value of 0.901, and Bartlet’s sphericity test (*p* < 0.001) confirmed sampling adequacy for EFA. The extracted ten factors were based on a scree plot and eigenvalues > 1.00. A 10-factor structure explained about 57% of the total variance. Factor loadings based on rotated factor matrix and explained variance of each factor were reported in Table 2. The factor loading of all items ranged from 0.464 to 0.807 on their corresponding factor.

**Table 2:** Factor loadings of the body image scale for youth (BISY) items based on rotated factor matrix

Items	Factors									
	1	2	3	4	5	6	7	8	9	10
*Emotions and behaviours*										
I have been humiliated or teased by others (family members, friends, teachers, and others in the community) because of my appearance and physical problems	**0.523**	0.245	− 0.036	0.094	0.044	0.134	− 0.015	0.161	0.129	− 0.226
I have been envious of seeing beautiful or well-built body individuals	**0.575**	0.108	0.156	0.042	0.376	− 0.009	0.050	0.086	0.088	0.194
I have felt ashamed because of my appearance	**0.728**	0.137	0.128	0.108	0.191	0.011	0.088	0.034	0.028	0.066
I have felt frustrated when others criticize my appearance	**0.730**	0.086	0.230	0.073	0.144	0.053	− 0.002	0.081	0.080	0.049
I have felt disgusted in some parts of my body	**0.654**	0.140	0.146	0.079	0.161	− 0.019	0.160	− 0.031	0.043	0.288
I have felt sad because of dissatisfaction with some of my appearance features	**0.687**	0.105	0.185	0.134	0.173	0.033	0.164	− 0.035	0.078	0.252
I have been afraid and worried that others will not accept me for my appearance	**0.664**	0.064	0.180	0.020	0.100	0.049	0.082	0.147	0.036	0.117
As far as I can remember, I have not done some activities I liked (buying clothes or attending extracurricular classes) because of derogatory remarks or criticism of others about my appearance	**0.673**	0.131	0.034	− 0.015	− 0.011	0.082	− 0.032	0.133	0.058	− 0.199
As far as I can remember, dissatisfaction with my body has been a barrier to my physical activities	**0.570**	0.254	− 0.052	− 0.057	0.006	0.007	− 0.067	0.044	0.045	− 0.244
As far as I can remember because I do not like some of my appearance features, I have looked less in the mirror	**0.734**	0.171	0.038	− 0.003	0.039	0.092	0.056	− 0.083	− 0.021	− 0.225
As far as I can remember, I have refused to attend certain gatherings because of some of my appearance features (body, or facial features)	**0.771**	0.104	0.018	− 0.004	− 0.036	0.082	0.062	0.066	− 0.006	− 0.171
As far as I can remember because I do not like some parts of my body, I have tried to wear clothes that make those parts less visible	**0.693**	0.059	0.087	0.075	0.089	− 0.040	0.047	− 0.066	0.035	0.204
As far as I can remember, I was not satisfied with my photos and I edited them	**0.573**	0.008	0.175	0.081	0.132	− 0.007	0.101	0.005	0.068	0.314
As far as I can remember when others criticize or blame me for my appearance, I have been angry or started a fight	**0.672**	0.025	− 0.034	− 0.053	0.030	0.099	0.107	0.104	− 0.020	− 0.187
As far as I can remember, to get my body fitted, I have harmed my body by extreme dieting or doing intense exercise	**0.510**	0.074	− 0.112	− 0.025	0.116	0.046	0.148	− 0.014	− 0.030	− 0.275
*Body evaluation*										
Overall, I have a positive evaluation of my appearance	0.369	**0.493**	0.249	0.188	0.061	0.133	0.153	0.087	− 0.044	− 0.003
Overall, I have a positive assessment of my physical health	0.203	**0.720**	0.187	0.049	0.056	0.105	0.070	0.039	− 0.081	− 0.039
Overall, I have a positive evaluation of my physical ability	0.257	**0.707**	0.195	0.096	0.033	0.075	0.145	0.018	− 0.040	− 0.126
In general, I think my appearance looks good from the other's point of view	0.228	**0.600**	0.114	0.310	− 0.031	0.092	0.041	− 0.057	0.023	0.151
I think I have good physical health from the others’ point of view	0.105	**0.804**	− 0.049	0.156	0.027	0.109	0.055	− 0.109	− 0.008	0.099
I think I have good physical ability from the others’ point of view	0.177	**0.782**	0.033	0.121	− 0.010	0.032	0.055	− 0.116	− 0.023	0.068
*Personal characteristics and strategies*										
I love myself, so I do not blame myself after hearing others criticize my appearance	0.302	0.231	**0.541**	0.163	0.116	0.059	0.149	0.062	0.022	0.106
Criticizing or praising my appearance by others does not affect my self-esteem	0.202	0.080	**0.732**	0.099	− 0.005	0.048	0.074	0.104	0.060	0.146
I have set goals for myself, so others' criticism of my appearance does not upset me, because I have more important goals that I think about more	0.249	0.068	**0.693**	0.167	− 0.012	0.122	0.302	0.170	− 0.004	0.107
When others criticize my appearance or give me a negative comment, I do not care about their comments	0.186	0.067	**0.562**	0.129	0.048	0.062	0.048	0.209	0.001	0.062
I do not allow others to judge or comment on my appearance	− 0.032	0.059	**0.620**	− 0.043	− 0.010	0.052	0.017	− 0.199	0.019	− 0.027
I do not mention my appearance flaws in front of others so that they are not allowed to comment	− 0.059	0.108	**0.646**	− 0.011	0.182	− 0.028	0.119	− 0.400	− 0.038	− 0.070
*Appearance importance in the future*										
I believe that my facial features increase the chances of a proper marriage in the future	0.037	0.127	0.018	**0.792**	− 0.082	0.089	− 0.006	− 0.131	0.042	0.056
I believe that my body increases the chances of a proper marriage in the future	0.018	0.148	0.001	**0.782**	− 0.051	0.082	− 0.005	− 0.167	0.004	0.035
I believe that considering my appearance features, I have enough chance of getting hired in my favorite jobs	0.122	0.181	0.113	**0.663**	− 0.037	− 0.002	0.136	0.036	− 0.034	− 0.103
I believe that my appearance features increase the chances of my career success in the future	0.046	0.124	0.196	**0.736**	− 0.079	0.047	0.056	− 0.074	0.008	0.040
Social models										
Celebrities on social networks set the beauty standard for me	0.161	0.046	− 0.058	− 0.024	**0.661**	0.012	0.193	0.105	0.040	− 0.218
I like to have faces or bodies like actors, singers, or costume models	0.196	0.024	0.038	0.006	**0.712**	− 0.026	0.080	0.206	0.025	0.079
I like to have a body like athletes	− 0.003	− 0.067	0.066	− 0.081	**0.465**	− 0.046	− 0.209	0.086	0.085	0.064
Social networks such as Instagram and Telegram affect my satisfaction with my body	0.213	0.070	0.045	− 0.098	**0.703**	− 0.021	0.065	0.056	0.073	− 0.082
The number of likes or comments I get from others on social media affects how I feel about my appearance	0.234	0.040	0.094	− 0.098	**0.535**	0.031	0.069	0.220	− 0.017	− 0.275
*Perceived social support*										
When I'm sad about my appearance, my family members are not indifferent to my sadness and support me emotionally	0.177	0.126	0.080	0.094	0.026	**0.751**	0.035	− 0.111	− 0.006	0.150
My family helps me to modify my appearance defects (for example, weight loss) by taking the necessary measures	0.132	0.113	0.052	0.078	0.051	**0.807**	0.068	− 0.063	0.016	0.036
My family understands my frustration with my appearance problems	0.117	0.044	0.142	0.066	0.024	**0.778**	0.047	− 0.097	− 0.038	0.124
When I'm upset about my appearance or my body, my friends support me emotionally	− 0.056	0.095	− 0.030	− 0.002	− 0.172	**0.589**	0.136	0.141	− 0.024	− 0.061
*Priority of health and spiritually*										
I do not desire to change my God-given appearance features because it was according to God's will	0.189	0.064	0.171	0.141	0.101	− 0.006	**0.625**	0.074	0.025	0.275
Having a good personality and good morals is more important than having a beautiful appearance	0.118	0.114	0.107	− 0.040	− 0.054	0.095	**0.618**	0.101	− 0.019	− 0.052
I will not perform any cosmetic manipulations or surgeries that endanger my health	0.090	0.058	0.000	0.124	0.182	0.065	**0.709**	0.076	0.046	0.135
In my opinion, healthy body parts are more important than physical beauty	0.078	0.087	0.153	− 0.012	− 0.015	0.104	**0.702**	− 0.071	− 0.045	− 0.133
*Appearance importance in social interactions*										
I like to shine in any group of people and look better than everyone else	0.085	− 0.103	− 0.028	− 0.067	0.233	0.009	0.064	**0.713**	− 0.023	− 0.028
I try to improve my appearance so that others are more attracted to me and pay attention to me	0.094	− 0.001	0.038	− 0.201	0.293	− 0.061	0.098	**0.669**	0.012	− 0.013
My appearance is very important to me because in our society people pay a lot of attention to people's appearance	0.185	− 0.051	0.107	− 0.204	0.245	− 0.147	0.065	**0.573**	0.200	0.052
*Perceived cultural values*										
In our society, people are first judged by their appearance	0.085	− 0.055	0.045	− 0.049	− 0.002	− 0.061	0.003	0.090	**0.729**	0.133
Our society's culture is such that it encourages people to use cosmetics and perform cosmetic surgeries	0.055	− 0.029	0.039	0.043	0.077	− 0.056	0.023	0.031	**0.755**	0.091
It is common in our society to tease and ridicule the appearance of people	0.097	− 0.024	− 0.036	0.025	0.112	0.078	− 0.042	− 0.032	**0.781**	− 0.026
*Empowerment*										
I have access to a counselor or psychologist when I feel dissatisfied with my appearance	− 0.095	0.052	0.074	− 0.035	− 0.156	0.217	− 0.058	0.136	0.048	**0.598**
In addition to the core lessons, we are taught other skills at school, such as ways to boost self-confidence and how to deal with ridiculers	− 0.042	0.064	0.083	0.027	− 0.069	0.064	0.131	− 0.087	0.167	**0.570**
Explained variance	14.24	6.58	5.80	5.25	5.10	4.71	4.37	3.83	3.62	3.50

Bold values indicate the highest factor loadings of items for each factor (subscale of BISY)

The number of items, intra-class correlation coefficients (ICCs), and Cronbach’s alpha (α) for each subscale was reported in Table 3. The ICC and Cronbach’s alpha values for the overall scale were 0.805 and 0.896, respectively. The ICC values for subscales of BISY ranged from 0.753 to 0.990. Moreover, the range of Cronbach’s alpha values for BISY subscales was between 0.42 and 0.92. The Cronbach’s alpha value of 0.42 for the “empowerment” subscale showed poor internal consistency for this subscale; we could either remove the subscale or consider another grouping for items. We preferred to retain the empowerment-related items and arrange another grouping and assess the structural validity of the new structural model. Therefore, the possibility of merging these items with the existing subscales considering their meaning and concept similarity, the two items of the empowerment subscale were merged with the perceived social support subscale. After this modification, the structural validity of the new 9-factor structure model was assessed using the CFA and the internal consistency of the new subscale was checked. The findings of CFA showed acceptable fit for the 9-structure 52-item model as follows: χ^2^/*df* = 2.77, GFI = 0.85, IFI = 0.88, CFI = 0.87, RMSEA (90% CI) = 0.045 (0.044–0.047). Moreover, Cronbach's alpha value for the 6-item perceived social support subscale was 0.67.

**Table 3:** The intra-class correlation coefficients (ICCs) and Cronbach’s alpha (α) for subscales of the body image scale for youth (BISY)

Subscales	Number of items	ICC	α
Emotions and behaviors	15	0.983	0.92
Body evaluation	6	0.990	0.86
Personal characteristics and strategies	6	0.973	0.77
Appearance importance in the future	4	0.916	0.80
Social models	5	0.753	0.72
Perceived social support	4	0.940	0.76
Priority of health and spiritually	4	0.982	0.68
Appearance importance in social interactions	3	0.860	0.71
Perceived cultural values	3	0.919	0.67
Empowerment	2	0.765	0.42

### Body image in boys and girls

Table 4 shows the mean and standard deviation for subscale and total scores of the BISY. Girls had significantly higher BISY total scores compared to boys. Moreover, except for social models, perceived social support, empowerment, and body evaluation subscales, other subscale scores of the BISY were significantly different in boys and girls; except for appearance importance in the social interactions subscale, girls had significantly higher scores, compared to boys.

**Table 4:** Mean and standard deviation (SD) for subscale and total scores of the body image scale for youth (BISY) in boys and girls

Scores	Boys (n = 422)	Girls (n = 435)	*p* value*
Personal characteristics and strategies	24.8 ± 17.8	31.4 ± 22.5	**< 0.001**
Priority on health and spiritually	14.5 ± 16.9	21.6 ± 20.3	**< 0.001**
Appearance importance in the future	24.9 ± 22.1	34.5 ± 22.9	**< 0.001**
Appearance importance in social interactions	67.4 ± 24.8	60.7 ± 25.9	**< 0.001**
Perceived cultural values	76.6 ± 20.9	79.6 ± 20.3	**0.031**
Social models	48.2 ± 23.6	46.1 ± 24.8	0.213
Perceived social support	30.8 ± 23.9	33.1 ± 24.8	0.159
Empowerment	68.7 ± 28.2	71.5 ± 26.4	0.125
Body evaluation	16.9 ± 16.9	19.2 ± 17.9	0.055
Emotions and behaviors	18.9 ± 19.2	23.5 ± 19.1	**< 0.001**
BISY total score	31.3 ± 10.2	34.8 ± 12.7	**< 0.001**

Data in bold, indicate statistically significant p values (*p* < 0.05)

*p values were derived using t-test

**Data is shown as mean ± SD

### Body image in body weight status groups

The comparison of mean and standard deviation for subscale and total scores of the BISY in body weight status groups are presented in Table 5. In boys, there were no significant differences in BISY total score among different body weight status groups. However, there were significant differences in BISY total score among underweight, normal weight, overweight and obese girls. Based on findings of post hoc tests, the BISY total score was significantly lower in normal weight compared to overweight (*p* = 0.009), and obese (*p* = 0.012) girls. In terms of subscales, there were significant differences in appearance importance in future, empowerment, and body evaluation subscale scores among different body weight stats groups in boys. Further post-hoc tests indicated that normal-weight boys had lower scores in appearance importance in future subscale compared to obese boys (*p* = 0.011), and underweight boys had lower scores in the empowerment subscale compared to their normal-weight (*p* = 0.009) and obese (*p* = 0.007) counterparts, and normal-weight had significantly lower scores in the body evaluation subscale compared to obese boys (*p* = 0.003). In girls, there were significant differences in body evaluation and emotion and behaviors subscale scores among different body weight stats groups. Further post-hoc tests indicated that normal weight had significantly lower scores in the body evaluation subscale compared to obese girls (*p* = 0.044); furthermore, normal weight girls had lower scores in the emotions and behaviors subscale compared to both overweight (*p* < 0.001) and obese (*p* = 0.001) girls.

**Table 5:** Total and subscale scores of the body image scale for youth (BISY) in boys and girls by body weight status

	Underweight (n = 39)	Normal weight (n = 554)	Overweight (n = 142)	Obese (n = 122)	*p* value*
*Boys (n = 422)*					
Personal characteristics and strategies	23.5 ± 16.5	24.3 ± 17.4	25.1 ± 17.0	26.0 ± 20.0	0.889
Priority of health and spiritually	20.5 ± 18.1	15.8 ± 17.5	11.7 ± 15.6	12.7 ± 15.8	0.115
Appearance importance in the future	31.8 ± 23.6	22.4 ± 20.9	26.7 ± 22.9	29.4 ± 23.8	**0.038**
Appearance importance in social interactions	68.9 ± 28.2	68.4 ± 24.9	68.3 ± 23.6	63.6 ± 25.4	0.462
Perceived cultural values	78.8 ± 19.1	76.5 ± 20.8	76.8 ± 19.6	76.4 ± 23.2	0.986
Social models	49.5 ± 23.5	48.1 ± 24.3	51.8 ± 22.0	45.1 ± 23.1	0.337
Perceived social support	27.8 ± 23.3	32.0 ± 23.3	26.7 ± 23.2	31.6 ± 26.1	0.372
Empowerment	47.7 ± 22.9	70.3 ± 27.6	62.8 ± 28.2	71.9 ± 28.9	**0.009**
Body evaluation	20.8 ± 17.8	14.2 ± 14.6	18.2 ± 16.4	23.1 ± 21.4	**0.004**
Emotions and behaviors	16.2 ± 18.3	17.0 ± 18.0	21.4 ± 19.0	22.6 ± 22.2	0.068
BISY total score	31.1 ± 9.2	30.4 ± 9.7	31.9 ± 9.3	33.0 ± 12.3	0.291
*Girls (n = 435)*					
Personal characteristics and strategies	25.3 ± 24.3	31.3 ± 22.3	30.1 ± 19.0	39.8 ± 26.7	0.062
Priority of health and spiritually	22.1 ± 22.3	21.0 ± 20.0	23.0 ± 19.5	24.3 ± 22.8	0.746
Appearance importance in the future	38.0 ± 27.6	32.9 ± 22.0	38.3 ± 26.0	39.2 ± 20.0	0.160
Appearance importance in social interactions	61.9 ± 28.0	59.4 ± 26.2	63.8 ± 24.7	65.5 ± 23.5	0.407
Perceived cultural values	79.5 ± 28.7	78.7 ± 19.6	82.5 ± 20.7	81.9 ± 17.4	0.483
Social models	40.0 ± 23.6	45.4 ± 25.5	49.9 ± 23.1	50.4 ± 21.9	0.212
Perceived social support	36.4 ± 27.5	32.7 ± 23.9	33.0 ± 26.9	35.1 ± 27.2	0.845
Empowerment	71.4 ± 30.6	71.3 ± 25.6	72.0 ± 28.8	72.6 ± 26.9	0.992
Body evaluation	19.2 ± 21.0	17.4 ± 16.6	22.5 ± 16.5	28.8 ± 23.7	**0.013**
Emotions and behaviors	23.0 ± 20.6	20.1 ± 16.5	32.0 ± 19.0	38.7 ± 26.4	**< 0.001**
BISY total score	34.0 ± 14.3	33.2 ± 11.7	38.6 ± 12.0	42.8 ± 17.0	**< 0.001**

Data in bold, indicate statistically significant p values (*p* < 0.05)

*p values were derived using the ANOVA (analysis of variance) or Welch test

**Data is shown as mean ± SD

Body image correlations with disordered eating and self-assessed physical features

There were significant correlations between BISY total score and EAT-26 total score in both boys (r = 0.22, *p* < 0.001) and girls (r = 0.22, *p* < 0.001). In addition, BISY total score was significantly correlated with dieting and bulimia and food preoccupation subscales in boys (r = 0.17, *p* = 0.001; r = 0.29, *p* < 0.001, respectively) and girls (r = 0.21, *p* < 0.001; r = 0.34, *p* < 0.001, respectively). In terms of self-assessed physical features of body, BISY total score was significantly correlated with physical appearance (r = −0.45, *p* < 0.001 in boys and r = −0.62, *p* < 0.001 in girls), ability (r = −0.37, *p* < 0.001 in boys and r = −0.44, *p* < 0.001 in girls), and health (r = −0.33, *p* < 0.001 in boys and r = −0.50, *p* < 0.001 in girls).

## Discussion

The current study reported the development and psychometric properties of the BISY, a 52-item scale that can be applied for the assessment of body image and its related factors in adolescent boys and girls. The current findings support the validity and reliability of the BISY. Moreover, current findings indicated sex and body weight status were significantly associated with body image in adolescents. Girls had a more negative body image compared to boys, and overweight and obese girls had significantly more negative body image compared to their normal-weight counterparts.

The BISY can be applied as a valid and reliable instrument for the assessment of different aspects related to body image in adolescents. This 52-item 10-factor solution which has emerged in EFA can explain an acceptable amount of variance in body image (more than 50%) in participants. The factor entitled “Emotions and behaviors” with 15 items explained the highest variance followed by “Body evaluation” and “Personal characteristics and strategies” as the second and third factors. Results of assessing internal consistency and test–retest reliability for the whole scale (BISY), indicate high reliability of the scale for assessment of body image in youth. For BISY subscales, the ICC values exceeded the acceptable value of 0.5 [36]. In terms of internal consistency of subscales, Cronbach’s alpha coefficients for all subscales exceeded the acceptable value of 0.7 [35], except for “priority of health and spiritually”, “perceived cultural values”, and “empowerment” subscales. The Cronbach’s alpha coefficients of 0.68 and 0.67 for the first two mentioned subscales, were approximately near the acceptable threshold of 0.7. However, the value of 0.42 for the “empowerment” subscale showed poor internal consistency for this subscale. In such cases, it is more common to delete the relevant item in the subscale to increase the Cronbach's alpha. As there are only two items in this subscale, this approach could not be applied. As mentioned in the results, we decided to retain the related items and arranged another grouping, and then assess the structural validity of the new 9-factor structure model using the CFA. Findings of CFA showed an acceptable fit for the new 9-factor structure model. Moreover, Cronbach's alpha value for the new perceived social support subscale (α = 0.67) was very close to the acceptable threshold of 0.7.

Based on the findings of the current study, girls had significantly a more negative body image compared to boys. In line with the current findings, previous studies reported a more negative body image and lower levels of body satisfaction in females compared to males [4, 37, 38]. In this regard, findings of a qualitative study conducted among 12–20 years Iranian adolescents reported that most adolescent girls did not have positive feelings about their bodily changes during puberty which led them to feel nervous or ashamed [37]. Hence, in the current study, having a more negative body image in adolescent girls compared to boys could be a result of body image dissatisfaction due to pubertal changes and its negative influence on their psychological well-being.

In the current study, overweight and obesity were associated with a more negative body image in girls, but not in boys. These findings may be due to perceptions of different ideals concerning body shape and size by adolescent girls and boys [38–40]. In this regard, previous findings demonstrated that while girls tend to be dissatisfied with their bodies when their BMI is average or above average; boys tend to be dissatisfied with their bodies when they have a BMI either below or above average [39, 40]. In another study, girls with excessive weight were more likely to be dissatisfied with their bodies, compared to overweight boys [38]. In addition, previous studies in western societies have shown that, whereas most girls prefer a slim body shape and a smaller body size, most boys prefer a muscular body shape and a larger body size [41–43]. In agreement with the current findings, in a previous qualitative study conducted among adolescents residing in Tehran, some overweight/obese adolescent boys had a positive self-image rather than a negative one. This perception is rooted in beliefs such as having higher resistance to illness and physical blows and having a similar ability to compete in sports and physical activities compared to their normal weight counterparts [44]. This may explain why body image did not differ among overweight and obese boys compared to their normal-weight counterparts in the current study.

To the best of our knowledge, this is the first study that develop a comprehensive scale for assessment of body image in Iranian adolescents covering several aspects related to body image perceptions including social factors, personal characteristics and strategies, attitudes, evaluation, as well as emotions and behaviors. The limitations of this study should also be taken into consideration. First, we did not measure adolescents’ body weight and height, and the BMI was calculated using the self-reported data. Hence, some adolescents may misreport their weight and height which may result in misclassification of their BMI. Second, due to the cross-sectional nature of the current study, causal inferences about body image in relation to body weight status are not possible. Finally, participants of the current study were recruited from Tehran (an urban community); therefore, it is recommended to conduct similar study on adolescents residing in suburban and rural areas to replicate the current findings.

## Conclusion

In conclusion, the current findings indicated that the BISY can be used as a valid and reliable tool for the assessment of body image and its related factors among Iranian adolescents by researchers and healthcare professionals. This scale can help professionals to identify those adolescents who are susceptible to developing a negative body image and its related health consequences like eating disorders. Moreover, in the current study, girls had more negative body image compared to boys. Additionally, overweight and obese girls had more negative body images compared to their normal-weight counterparts. Therefore, it is recommended that body image be incorporated as an important component of future health promotion programs with a focus on adolescent girls as the target population, specifically in those interventions targeting overweight and obesity during adolescence.

## Supplementary Information

**Additional file 1.** The BISY and its scoring procedure.

## Data Availability

Data used in the current study would be available from the corresponding author on reasonable request.

## Abbreviations

BMI: Body mass index
CFA: Confirmatory factor analysis
CVI: Content validity index
CVR: Content validity ratio
EFA: Exploratory factor analysis
ICC: Intra-class correlation coefficient
BISY: Body image scale for youth
