# Supplementary material for: Development and validation of the body image scale for youth (BISY)
Source: J Eat Disord. 2022 Sep 6;10:136. doi: 10.1186/s40337-022-00657-z (PMC9450403; doi:10.1186/s40337-022-00657-z)
Supplement: Supplementary file 1 — Additional file 1. The BISY and its scoring procedure. [file 40337_2022_657_MOESM1_ESM.docx]

**Supplementary table 1. The BISY and its scoring procedure**

| No. | **Items** | **Likert points** | | | | |
| --- | --- | --- | --- | --- | --- | --- |
|  |  | Completely agree | Somewhat agree | Neither agree nor disagree | Somewhat disagree | Completely disagree |
| Personal characteristics and strategies | | | | | | |
| 1 | I love myself, so I do not blame myself after hearing others criticize my appearance. | 1 | 2 | 3 | 4 | 5 |
| 2 | Criticizing or praising my appearance by others does not affect my self-esteem. | 1 | 2 | 3 | 4 | 5 |
| 3 | I have set goals for myself, so others' criticism of my appearance does not upset me, because I have more important goals that I think about more. | 1 | 2 | 3 | 4 | 5 |
| 4 | When others criticize my appearance or give me a negative comment, I do not care about their comments. | 1 | 2 | 3 | 4 | 5 |
| 5 | I do not allow others to judge or comment on my appearance. | 1 | 2 | 3 | 4 | 5 |
| 6 | I do not mention my appearance flaws in front of others so that they are not allowed to comment. | 1 | 2 | 3 | 4 | 5 |
| Priority of health and spirituality | | | | | | |
| 7 | I do not desire to change my God-given appearance features because it was according to God's will. | 1 | 2 | 3 | 4 | 5 |
| 8 | Having a good personality and good morals is more important than having a beautiful appearance. | 1 | 2 | 3 | 4 | 5 |
| 9 | I will not perform any cosmetic manipulations or surgeries that endanger my health. | 1 | 2 | 3 | 4 | 5 |
| 10 | In my opinion, healthy body parts are more important than physical beauty. | 1 | 2 | 3 | 4 | 5 |
| Appearance importance in the future | | | | | | |
| 11 | I believe that my facial features increase the chances of a proper marriage in the future. | 1 | 2 | 3 | 4 | 5 |
| 12 | I believe that my body increases the chances of a proper marriage in the future. | 1 | 2 | 3 | 4 | 5 |
| 13 | I believe that considering my appearance features, I have enough chance of getting hired in my favorite jobs. | 1 | 2 | 3 | 4 | 5 |
| 14 | I believe that my appearance features increase the chances of my career success in the future. | 1 | 2 | 3 | 4 | 5 |
| Appearance importance in social interactions | | | | | | |
| 15 | I like to shine in any group of people and look better than everyone else. | 5 | 4 | 3 | 2 | 1 |
| 16 | I try to improve my appearance so that others are more attracted to me and pay attention to me. | 5 | 4 | 3 | 2 | 1 |
| 17 | My appearance is very important to me because in our society people pay a lot of attention to people's appearance. | 5 | 4 | 3 | 2 | 1 |
| Social models | | | | | | |
| 18 | Celebrities on social networks set the beauty standard for me. | 5 | 4 | 3 | 2 | 1 |
| 19 | I like to have faces or bodies like actors, singers, or costume models. | 5 | 4 | 3 | 2 | 1 |
| 20 | I like to have a body like athletes. | 5 | 4 | 3 | 2 | 1 |
| 21 | Social networks such as Instagram and Telegram affect my satisfaction with my body. | 5 | 4 | 3 | 2 | 1 |
| 22 | The number of likes or comments I get from others on social media affects how I feel about my appearance. | 5 | 4 | 3 | 2 | 1 |
| Perceived cultural values | | | | | | |
| 23 | In our society, people are first judged by their appearance. | 5 | 4 | 3 | 2 | 1 |
| 24 | Our society's culture is such that it encourages people to use cosmetics and perform cosmetic surgeries. | 5 | 4 | 3 | 2 | 1 |
| 25 | It is common in our society to tease and ridicule the appearance of people. | 5 | 4 | 3 | 2 | 1 |
| Perceived social support | | | | | | |
| 26 | When I'm sad about my appearance, my family members are not indifferent to my sadness and support me emotionally. | 1 | 2 | 3 | 4 | 5 |
| 27 | My family helps me to modify my appearance defects (for example, weight loss) by taking the necessary measures. | 1 | 2 | 3 | 4 | 5 |
| 28 | My family understands my frustration with my appearance problems. | 1 | 2 | 3 | 4 | 5 |
| 29 | When I'm upset about my appearance or my body, my friends support me emotionally. | 1 | 2 | 3 | 4 | 5 |
| Empowerment | | | | | | |
| 30 | I have access to a counselor or psychologist when I feel dissatisfied with my appearance. | 1 | 2 | 3 | 4 | 5 |
| 31 | In addition to the core lessons, we are taught other skills at school, such as ways to boost self-confidence and how to deal with ridiculers. | 1 | 2 | 3 | 4 | 5 |
| Body evaluation | | | | | | |
| 32 | Overall, I have a positive evaluation of my appearance. | 1 | 2 | 3 | 4 | 5 |
| 33 | Overall, I have a positive assessment of my physical health. | 1 | 2 | 3 | 4 | 5 |
| 34 | Overall, I have a positive evaluation of my physical ability. | 1 | 2 | 3 | 4 | 5 |
| 35 | In general, I think my appearance looks good from the other's point of view. | 1 | 2 | 3 | 4 | 5 |
| 36 | I think I have good physical health from the others’ point of view. | 1 | 2 | 3 | 4 | 5 |
| 37 | I think I have good physical ability from the others’ point of view. | 1 | 2 | 3 | 4 | 5 |
|  |  | **Likert points** | | | | |
|  |  | Always | Often | Sometimes | Seldom | Never |
| Emotions and behaviors | | | | | | |
| 38 | I have been humiliated or teased by others (family members, friends, teachers, and others in the community) because of my appearance and physical problems. | 5 | 4 | 3 | 2 | 1 |
| 39 | I have been envious of seeing beautiful or well-built body individuals. | 5 | 4 | 3 | 2 | 1 |
| 40 | I have felt ashamed because of my appearance**.** | 5 | 4 | 3 | 2 | 1 |
| 41 | I have felt frustrated when others criticize my appearance. | 5 | 4 | 3 | 2 | 1 |
| 42 | I have felt disgusted in some parts of my body. | 5 | 4 | 3 | 2 | 1 |
| 43 | I have felt sad because of dissatisfaction with some of my appearance features. | 5 | 4 | 3 | 2 | 1 |
| 44 | I have been afraid and worried that others will not accept me for my appearance. | 5 | 4 | 3 | 2 | 1 |
| 45 | As far as I can remember, I have not done some activities I liked (buying clothes or attending extracurricular classes) because of derogatory remarks or criticism of others about my appearance. | 5 | 4 | 3 | 2 | 1 |
| 46 | As far as I can remember, dissatisfaction with my body has been a barrier to my physical activities. | 5 | 4 | 3 | 2 | 1 |
| 47 | As far as I can remember because I do not like some of my appearance features, I have looked less in the mirror. | 5 | 4 | 3 | 2 | 1 |
| 48 | As far as I can remember, I have refused to attend certain gatherings because of some of my appearance features (body, or facial features). | 5 | 4 | 3 | 2 | 1 |
| 49 | As far as I can remember because I do not like some parts of my body, I have tried to wear clothes that make those parts less visible. | 5 | 4 | 3 | 2 | 1 |
| 50 | As far as I can remember, I was not satisfied with my photos and I edited them. | 5 | 4 | 3 | 2 | 1 |
| 51 | As far as I can remember when others criticize or blame me for my appearance, I have been angry or started a fight. | 5 | 4 | 3 | 2 | 1 |
| 52 | As far as I can remember, to get my body fitted, I have harmed my body by extreme dieting or doing intense exercise. | 5 | 4 | 3 | 2 | 1 |
